# Demographic and environmental factors associated with disability in India, Laos, and Tajikistan: a population-based cross-sectional study

**DOI:** 10.1186/s12889-022-12846-1

**Published:** 2022-03-29

**Authors:** Michael Zhu Chen, Lindsay Lee, Carolina Fellinghauer, Alarcos Cieza, Somnath Chatterji

**Affiliations:** 1 Sensory Functions, Disability and Rehabilitation, Department of Noncommunicable Diseases, World Health Organization, Avenue Appia 20, 1211 Geneva, Switzerland; 2 Department of Data and Analytics, World Health Organization, Avenue Appia 20, 1211 Geneva, Switzerland

**Keywords:** Disability, Capacity, Functioning, Demographic, Environment, Model Disability Survey, India, Laos, Tajikistan

## Abstract

**Background:**

The number of people experiencing functional limitations due to health conditions (capacity) is expected to increase in low and middle-income countries as populations age and rates of non-communicable disease rise. This trend could raise the prevalence and levels of disability worldwide. Understanding the demographic and environmental factors associated with disability can inform the design of policy interventions to make societies more accessible and inclusive for all.

**Methods:**

Approximately 2,500–3,000 participants in each of India, Laos, and Tajikistan responded to the Gallup World Poll and the World Health Organization’s Brief Model Disability Survey through face-to-face interviews. For each country, random forest regression was performed to explore the associations of demographic and environmental factors with disability while controlling for capacity. Using the variable importance measures generated by the random forest models, linear regression models were built in a stepwise manner for each country to predict disability level based on these contextual factors.

**Results:**

Capacity was strongly associated with disability in all three countries. Most of the variance in disability was explained by minimally adjusted linear models that included only capacity, sex, and age. Inclusion of additional demographic factors and environmental factors explained slightly more of the variance in disability score. Across all three countries, the level of basic infrastructure, public services, and financial stability were moderately associated with disability. Age, sex, employment status, the use of assistive technologies, and other factors had associations with disability that were highly variable across countries.

**Conclusion:**

While capacity was the main determinant of disability, individual demographic and environmental factors were associated with disability in a country-specific manner while controlling for the effects of capacity.

**Supplementary Information:**

The online version contains supplementary material available at 10.1186/s12889-022-12846-1.

## Background

The 2011 World Report on Disability by the World Health Organization (WHO) found that over 1 billion people—15% of the world’s population—experience significant disability [1]. People with disability tend to have worse access to healthcare and poorer health outcomes [2]. By signing the Convention on the Rights of Persons with Disabilities (CRPD) and the United Nations Sustainable Development Goals, countries have made commitments to ensure that people with disability are allowed to participate in society on an equal basis with others [3, 4].

According to the WHO International Classification of Functioning, Disability, and Health (ICF), the definition of disability is a reduction in everyday functioning that is the outcome of an interaction between an individual’s capacity and that individual’s personal and environmental factors [5]. Health conditions lead to reduced body capacity in varying degrees depending on the environment in which a person lives. According to this understanding, disability cannot only be described using a binary categorization of “disabled” or “not disabled” based on certain ad hoc or arbitrary criteria. Rather, disability should be understood as a spectrum ranging from no disability to severe disability.

There remains a great need for nationally representative, comprehensive, relevant, and standardized data on the demographic and environmental factors that affect disability. Little is known about which factors are associated with disability in low and middle income settings as well as the relative strength of these associations. The 2011 World Report on Disability reported the global prevalence of disability among adults as 11.8% in higher income countries and 18.0% in lower income countries [1] but did not include comprehensive information on the demographic and environmental factors that may be associated with disability. Few studies have collected standardized, nationally-representative data on disability in tandem with these contextual factors at the individual level in LMICs; one study reported that accessible community spaces to socialize, transportation systems, personal assistance, and regular medication use were associated with disability in Cambodia [6]. A study in Cameroon found that a person’s dwelling, access to transportation, and other aspects of the physical environment had the highest influence on disability [7]. A greater understanding of the relevance of these factors for disability is essential to create policies that 1) reduce disparities for people with disability across demographic and environmental contexts and 2) make environments more accessible and inclusive for all. There is an especially urgent need to understand how demographics and the environment affect disability in LMICs, which are experiencing demographic and epidemiological transitions that will likely increase the prevalence of chronic health conditions in the coming decades [1, 6, 8–10].

This study aims to identify demographic and environmental factors that are most strongly associated with disability in India, Tajikistan, and Laos through a joint administration of the Brief Model Disability Survey (MDS) and the Gallup World Poll. These findings will provide much-needed evidence about disability in LMICs that will expand our understanding of the contextual factors contributing to disability in these settings, highlight disparities in disability across groups in the population, and inform multi-level individual, community, and public policy-based approaches that will enable people to be equal participants in society regardless of their level of disability.

## Methods

### Study design

The study was a cross-sectional survey conducted in India, Tajikistan, and Laos.

### Model disability survey

The Model Disability Survey (MDS) was created by the WHO and the World Bank in 2011 to measure disability in a standardized and comprehensive fashion [11]. The MDS follows the ICF’s definition of disability: it captures information about an individual’s local environment, everyday functional capacity associated with health conditions alone (a “capacity” score), and actual everyday functioning taking into account respondents’ health and the environment (a “disability” score). The MDS was designed to be administered to a general population sample without pre-screening of participants in order to understand the distribution of the population on the disability continuum. The MDS has been piloted, validated, and implemented on a national and regional scale in countries around the world, including Afghanistan, Cameroon, Chile, Costa Rica, Pakistan, the Philippines, and Sri Lanka [12]. For this study, the core modules of the MDS were condensed into a brief version (Brief MDS or B-MDS), which can be administered as a short ten- to fifteen-minute disability module as a part of other population surveys [13].

### Data collection

Data were collected using an integrated B-MDS & Gallup World Poll survey that was administered through face-to-face interviews in India, Laos, and Tajikistan in 2018. The B-MDS survey questions are provided in Supplemental Methods 1 and the Gallup World Poll survey questions can be found in the Gallup Worldwide Research Methodology and Codebook [14]. The Gallup World Poll is an annual survey administered by Gallup Inc. in over 150 countries around the world that captures self-reported information about the environments in which people live, including financial status, public safety, and community infrastructure & services [14]. Gallup translated the surveys and conducted the interviews in a culturally-sensitive manner.

Gallup cleaned the dataset and computed the Gallup indices based on the responses. The Gallup indices were computed on a scale of 0 to 100, where 100 is the most favorable. Gallup also provided sampling weights to correct for sampling bias (such as non-response bias) and to ensure that the data were nationally-representative with respect to the demographics of the country. A detailed description of Gallup’s sampling, data collection, and data processing methodology can be found in the Gallup Worldwide Research Methodology and Codebook [14].

### Participants

There were 3,000 participants in India, 3,000 participants in Tajikistan, and 2,504 participants in Laos. The sampling frame was non-institutionalized people at least 15-years-old. Gallup used a stratified, multistage cluster sampling method to select participants in a nationally-representative manner [14]. The study size was chosen based on prior population surveys conducted by Gallup, prior studies using the MDS, and practical limitations of time and cost.

### Variables

The main outcome variable was the disability score, which measures the level of functioning due to the interaction between health conditions and contextual factors. The predictor variables included capacity score (a measure of everyday functioning as a direct consequence of health conditions), assistive technology use, demographic factors including age, sex, marital status, education, income, urban/rural setting, and employment status, and a variety of environmental factors from the Gallup Worldwide Research Methodology and Codebook [14] (such as financial life, food and shelter, law and order, and civic engagement). Some of the Gallup indices were excluded due to missing data, redundancy, or collinearity (Supplementary Methods 2).

### Bias

The main sources of bias are sampling bias, self-reporting bias, and differences in perspectives on disability across countries and cultures. Sampling bias was addressed by Gallup’s nationally-representative, stratified, multistage cluster sampling approach. Self-reporting bias is inherent due to the survey-based nature of the study. Gallup’s culturally-sensitive translations and in-person interviews helped reduce the bias from the differences in views of disability across countries and cultures.

### Software

RStudio [15] and R [16] were used to perform all analyses. Publicly available R packages were used for some analyses and are noted in the relevant sections below.

### Research ethics

Approval for all aspects of the study, including data collection and analysis, was obtained from the WHO Research Ethics Review Committee (Protocol ID: 0,003,040).

### Rasch analysis

Rooted in item response theory, Rasch analysis is a method that enables the generation of an interval-scaled score for a latent variable of interest based on the distribution of responses to a set of survey questions [17]. In the course of previous analysis of MDS data, Rasch analysis was performed using the WHOMDS package [18] to calculate a capacity score for each participant. Participants across all three countries were pooled when generating the capacity scores to ensure that scores were comparable between the three countries. Scores were not computed if a participant had two or more missing values for the items used to generate the scales. Rasch analysis was used to generate disability scores in the same manner. Disability scores were also comparable across countries. However, the capacity score and disability score cannot be directly compared because they were generated by separate Rasch analyses. A higher capacity or disability score indicates greater difficulty with everyday functioning. Disability scores were used to assign categories of disability (No, Mild, Moderate, and Severe) and create density plots for each country based on the following cutoffs: mean—1 standard deviation, mean, mean + 1 standard deviation.

### Variable selection and cleaning

A standard set of demographic variables (including age, sex, educational level, income quintiles, living in a large city vs a suburban/rural setting, and employment status) was included in the analysis. Age was coded in categories of 15–29, 30–49, and 50 or above. Educational level was coded in categories of primary education or less, secondary education, and tertiary education or more. Employment status was a categorical variable with six categories: unemployed (baseline category), employed full time for an employer, employed full time for self, employed part time and do not want full time, employed part time and want full time, and out of the workforce. Data on the presence/absence of health conditions were obtained from the MDS.

Environmental factors were measured by the Gallup indices. Gallup indices were excluded from the analysis if the data for an index were entirely missing for one or more countries. Indices were also excluded if two or more indices were similar, in which case only the most comprehensive and relevant index was included in the analysis in order to avoid multicollinearity. Additional information about the excluded Gallup indices is provided in Supplemental Methods 2.

### Descriptive analysis

A descriptive table with summary statistics for each country’s participants was generated using the arsenal package [19]. To compare values between countries, analysis of variance (ANOVA) for continuous variables or Pearson’s Chi-squared test for binary and categorical variables was performed and the associated *p*-values were reported. For continuous variables, the mean and standard deviation were reported for variables that were approximately normally distributed, while the median and inter-quartile range (IQR) were reported for variables that were not normally distributed.

### Random forest regression

Random forest regression was performed separately for each country in order to explore potential associations between the input variables and the disability score in a hypothesis-free manner. The randomForest package [20] was used to fit the random forest models. The inputs were capacity score, demographic factors, and environmental factors. The outcome was the disability score. A total of 5,000 trees were used, and 6 variables were tried at each split. Sampling weights were not used in the random forest regression. For each variable, the variable importance measure (VIM) was obtained by computing the increase in mean squared error when the variable values were randomly shuffled. VIM values were used to rank the importance of each input variable in relation to the disability score.

Next, these fitted random forest models were analyzed to identify pair-wise interactions between input variables using the forestFloor package [21]. The identified candidate interactions were then included as potential regressors in subsequent linear regression modelling. First, forestFloor was used to generate individual two-dimensional feature contribution plots, which represent the change in the disability score due to the marginal contribution from a single input variable. The individual feature contribution plots were color-coded according to variables that may be involved in an interaction. Potential interactions were visually identified from these plots based on color patterns and quality of fit (*R*^*2*^ values from a *k*-nearest neighbor analysis). These potential interactions were then plotted in a three-dimensional combined feature contribution plot, which showed the joint contribution of two variables to the disability score. Feature contribution plots were systematically reviewed by visual inspection and the goodness-of-fit using a *k*-nearest neighbor surface to identify candidate interactions. An interaction was likely if there was a high-quality fit in the combined feature contribution plot compared to the individual feature contribution plots for a given pair of variables. Quality of fit was assessed by visual inspection and by comparing the *R*^*2*^ value for the combined features to the *R*^*2*^ values for the individual features. The identified candidate interactions were subsequently included in the stepwise construction of linear regression models.

### Stepwise linear regression

For each country, stepwise linear regression was performed to quantify the strength of associations between the capacity score, demographic factors, and environmental factors with the disability score. Sampling weights were used to weight the residuals when fitting the regression in order to ensure that the fitted models were nationally representative. First, a minimum-parameter linear model was fit: the regressors were age, sex, and capacity score and the outcome variable was disability score. Capacity score was included in the minimum-parameter model since health conditions are known to be strongly associated with disability score a priori.

Using the stepAIC function in the MASS package [22], regressors (and any candidate interactions identified by random forest regression) were sequentially added to the model based on the Akaike information criterion (AIC) of the resulting model. The AIC is computed using the following formula: $2k-\mathrm{ln}(L)$, where $k$ is the number of parameters in the model and $\mathrm{ln}(L)$ is the log likelihood of the model. Regressors were retained in the stepwise model if they reduced the AIC, which indicates that the variable added significantly more explanatory power to the model. For additional verification, the likelihood ratio test (*F*-test) was used to compare the goodness-of-fit for the final stepwise model and minimum-parameter model.

The beta coefficients of the final models were used to assess the strengths of the associations between the regressors and disability in each country and to compare these associations between different countries. A *p*-value for each beta coefficient was calculated using the *t*-test.

Multi-level regression was not performed because the within-country geographic location of participants was not recorded, the study was not designed with regional-level representativeness in mind, and a multi-level approach would reduce power to detect associations between contextual factors and disability by controlling for baseline differences in disability across different regions.

### Missing or incomplete data

Missing data were imputed for each participant based on the mean or mode value for the variable in their home country. The mean was used for continuous variables and the mode was used for categorical variables.

### Sensitivity analysis

To ensure that the imputation of missing values did not significantly affect the results, the analysis was repeated with the exclusion of participants that had any missing values. The results were compared with the results of the main analysis to identify any significant deviations.

## Results

### Descriptive analysis

A total of 8,504 participants were surveyed, with 3,000 in India, 2,504 in Laos, and 3,000 in Tajikistan. Imputation was performed for 256 participants, of which 187 were missing just one value, 33 were missing two values, and one participant was missing three values, out of a total of 25 variables. Unweighted summary statistics for the samples in each of the three countries are presented in Table 1. Since these data are unweighted, the variables in Table 1 reflect the raw baseline characteristics of the sample and are not fully representative of national demographics. The demographic variables and Gallup indices were fairly similar between India, Laos, and Tajikistan. However, Tajikistan differed from India and Laos on certain measures. For instance, much more of Tajikistan’s population resided in rural areas and many more people were out of the workforce. Participants in Tajikistan were also more educated on average compared to participants in India and Laos.

**Table 1:** Baseline characteristics of participants in India, Laos, and Tajikistan

**Variable**	**India** (*N* = 3000)	**Laos** (*N* = 2504)	**Tajikistan** (*N* = 3000)	***p-*value**
**Demographic Factors**				
**Sex**				< 0.001
Male	1721 (57.4%)	1151 (46.0%)	1173 (39.1%)	
Female	1279 (42.6%)	1353 (54.0%)	1827 (60.9%)	
**Age**				0.001
15–29	996 (33.2%)	748 (29.9%)	1042 (34.7%)	
30–49	1259 (42.0%)	1086 (43.4%)	1179 (39.3%)	
50 or above	745 (24.8%)	670 (26.8%)	779 (26.0%)	
**Marital status**				0.098
Not currently married	821 (27.4%)	640 (25.6%)	843 (28.1%)	
Married	2179 (72.6%)	1864 (74.4%)	2157 (71.9%)	
**Urban/rural setting**				0.039
Lives in a rural area, small town/village, or suburb	2760 (92.0%)	2256 (90.1%)	2744 (91.5%)	
Lives in a large city	240 (8.0%)	248 (9.9%)	256 (8.5%)	
**Per Capita Income Quintiles**				0.079
0-20%	510 (17.0%)	493 (19.7%)	504 (16.8%)	
20-40%	582 (19.4%)	473 (18.9%)	544 (18.1%)	
40-60%	606 (20.2%)	489 (19.5%)	587 (19.6%)	
60-80%	605 (20.2%)	497 (19.8%)	628 (20.9%)	
80-100%	697 (23.2%)	552 (22.0%)	737 (24.6%)	
**Education Level**				< 0.001
Completed primary or less	1823 (60.8%)	1515 (60.5%)	644 (21.5%)	
Completed secondary	952 (31.7%)	755 (30.2%)	1628 (54.3%)	
Completed tertiary	225 (7.5%)	234 (9.3%)	728 (24.3%)	
**Employment Status**				< 0.001
Unemployed	135 (4.5%)	7 (0.3%)	171 (5.7%)	
Employed full time for an employer	872 (29.1%)	362 (14.5%)	515 (17.2%)	
Employed full time for self	370 (12.3%)	1087 (43.4%)	110 (3.7%)	
Employed part time and do not want full time	257 (8.6%)	537 (21.4%)	268 (8.9%)	
Employed part time and want full time	301 (10.0%)	381 (15.2%)	299 (10.0%)	
Out of the workforce	1065 (35.5%)	130 (5.2%)	1637 (54.6%)	
**Environmental Factors**				
**Community Basics Index**	75.4 (26.9)	69.0 (25.5)	83.6 (19.4)	< 0.001
**Civic Engagement Index**	32.6 (36.2)	27.9 (28.8)	36.6 (31.0)	< 0.001
**Communications Access Index**	54.2 (31.6)	53.9 (35.3)	54.2 (33.2)	0.936
**Financial Life Index**	36.2 (26.8)	37.0 (20.5)	59.7 (30.6)	< 0.001
**Food and Shelter Index**	62.4 (42.3)	44.6 (41.8)	62.9 (41.2)	< 0.001
**Law and Order Score**	77.7 (26.8)	72.3 (32.7)	91.0 (20.1)	< 0.001
**Social Life Index**	71.8 (33.5)	77.6 (29.7)	85.1 (26.3)	< 0.001
**Youth Development Index**	81.7 (28.4)	80.6 (27.8)	91.1 (19.8)	< 0.001
**MDS Variables**				
** Capacity Score**	9.9 (0.0, 37.8)	37.8 (24.2, 48.3)	9.9 (0.0, 31.1)	< 0.001
** Disability Score**	28.0 (1.4, 45.3)	41.5 (30.9, 49.5)	11.3 (1.4, 35.0)	< 0.001
** Assistive Technology (AT)**				< 0.001
Do not use AT and do not need any AT	2131 (71.0%)	1636 (65.3%)	2155 (71.8%)	
Use AT and do not need more AT	556 (18.5%)	414 (16.5%)	570 (19.0%)	
Need more AT	313 (10.4%)	454 (18.1%)	275 (9.2%)	
**Health Conditions**				
Vision Loss	263 (8.8%)	373 (14.9%)	299 (10.0%)	< 0.001
Hearing Loss (partial or total)	148 (4.9%)	204 (8.1%)	114 (3.8%)	< 0.001
Heart disease (coronary heart disease, heart attack)	92 (3.1%)	236 (9.4%)	303 (10.1%)	< 0.001
Stroke	66 (2.2%)	43 (1.7%)	48 (1.6%)	0.192
Diabetes	123 (4.1%)	97 (3.9%)	76 (2.5%)	0.002
Arthritis	291 (9.7%)	389 (15.5%)	379 (12.6%)	< 0.001
Bronchitis or emphysema	113 (3.8%)	100 (4.0%)	113 (3.8%)	0.884
Asthma or allergic respiratory disease	157 (5.2%)	245 (9.8%)	206 (6.9%)	< 0.001
Back pain	656 (21.9%)	1224 (48.9%)	1067 (35.6%)	< 0.001
Depression	371 (12.4%)	305 (12.2%)	419 (14.0%)	0.084
Anxiety	612 (20.4%)	511 (20.4%)	371 (12.4%)	< 0.001
Trauma	153 (5.1%)	243 (9.7%)	111 (3.7%)	< 0.001

For the capacity and disability scores, the median (first quartile, third quartile) is shown. For categorical and binary variables, the number in each category (percentage of total participants) is shown. For the environmental factors, the mean (standard deviation) is shown. Sampling weights were not used to adjust these values, so this table should be interpreted as representing the characteristics of the sample but not of the countries themselves

**Fig. 1:**
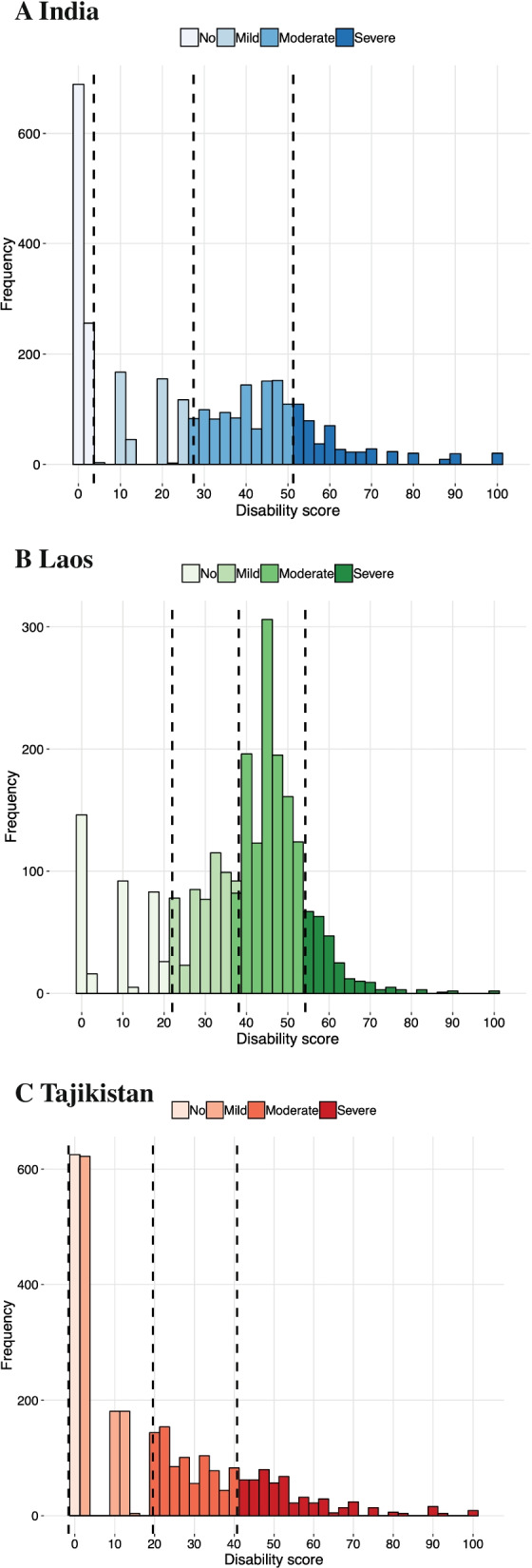
Distribution of disability score for (**A**) India, (**B**) Laos, and (**C**) Tajikistan. Cutoffs between levels of No, Mild, Moderate, and Severe disability are based on the mean—1 standard deviation, mean, and mean + 1 standard deviation for each country

The capacity score is a measure of an individual’s level of everyday functioning only as a direct consequence of their health condition, while the disability score is a measure of an individual’s everyday functioning due to the interaction between their health condition and contextual factors (taking outside help and other environmental factors into account). For both scores, a higher score indicates a higher degree of disability and reduced functioning. The median capacity score in Laos (37.8) was much higher than in India (9.9) and Tajikistan (9.9), indicating that participants in Laos tended to experience more difficulties in everyday functioning as a direct consequence of their health conditions. The mean disability score in Laos (41.5) was also higher than in India (28.0) and Tajikistan (11.3). The distributions of disability score for each country revealed that there was a wide range of disability within each country (Fig. 1). Laos had more people who experienced moderate levels of disability, while India and Tajikistan had fairly similar distributions (Fig. 1). The demographic and environmental factors varied between countries, although some factors, such as assistive technology (AT) use, were fairly similar (Table 1).

### Random forest regression

Random forest regression models trained on capacity score and all demographic & environmental factors for each country captured an average of 59.3% of the variance in disability score across countries (Table S1). Capacity score was the most important predictor of disability score by a large margin. Other than capacity score, the Community Basics Index had relatively high importance in all three countries. An age of 50 or above was a relatively important predictor of disability in Tajikistan and Laos but was less important in India. The other variables were ranked less consistently between the three countries, indicating that there was substantial heterogeneity in the associations between these variables and disability across the three countries.

Next, the fitted random forest models were used to identify candidate interactions for inclusion in subsequent linear regression modelling. Individual feature contribution plots (examples shown in Figure S1) and combined feature contribution 3D plots (example in Figure S2) were used to identify three to six candidate interactions for each country (Table 2). These interactions were subsequently included in the set of candidate regressors for stepwise construction of linear regression models.

**Table 2:** Candidate interactions identified from the random forest models. *R*^*2*^ values are shown for the individual feature contribution plots and the combined feature contribution plot

**Country**	**Variable 1**		**Variable 2**		**Interaction**
	**Name**	***R***^***2***^** value**	**Name**	***R***^***2***^** value**	***R***^***2***^** value**
**India**	Capacity Score	0.98	Employed part time and want full time	0.57	0.97
	Age 50 or above	0.94	Out of the workforce	0.03	0.88
	Community Basics Index	0.84	Youth Development Index	0.91	0.89
**Laos**	Capacity Score	0.98	Community Basics Index	0.54	0.98
	Capacity Score	0.98	Living in big city	0.68	0.98
	Per capita income quintiles	0.79	Have AT and does not need more	0.61	0.81
	Per capita income quintiles	0.79	Marital status	0.33	0.79
	Living in big city	0.68	Have AT and does not need more	0.61	0.63
	Living in big city	0.68	Financial Life Index	0.68	0.7
**Tajikistan**	Capacity Score	0.95	Civic Engagement Index	0.32	0.95
	Capacity Score	0.95	Living in big city	0.36	0.95
	Capacity Score	0.95	Age 50 or above	0.97	0.98

### Stepwise linear regression

In the minimum-parameter linear regression model for India, the disability score increased by 0.833 (95% CI: 0.807 to 0.860) points for each additional point of capacity score (Table S2), all else being equal. Sex and age were not associated with disability in this model.

The stepwise addition of demographic and environmental factors to the minimum-parameter model for India resulted in a final linear model that had a better fit to the data based on the likelihood ratio test (*F* = 7.8227, *p* < 0.001). In this final model, the association between capacity and disability was slightly attenuated (Table 3). There was an average increase of 0.808 (95% CI: 0.779 to 0.838) points of disability score for each point increase in capacity score, all else being equal. Female sex was associated with an average increase of 1.542 (95% CI: 0.344 to 2.741) in disability score compared to being male. Higher (i.e. better) Youth Development, Civic Engagement, and Financial Life Indices were associated with a lower disability score. Being out of the workforce was associated with a disability score that was 2.161 (95% CI: 3.591 to 0.731) lower on average. Two interactions were retained in the final stepwise model. An age of 50 or above had a significant interaction with being out of the workforce, with a coefficient of 2.822 (95% CI: 0.221 to 5.423). The minimum-parameter and final stepwise models for India had *R*^*2*^ values of 0.581 and 0.591, indicating that including contextual factors increased the variance explained by 1.0%.

**Table 3:** Final linear model for disability after stepwise addition of input variables for India. *R*^*2*^ = 0.591

		**Confidence Interval**		
**Term**	**Value**	**Lower 95%**	**Upper 95%**	***p-*value**
(Intercept)	16.442	14.137	18.748	< 0.001
Capacity Score	0.808	0.779	0.838	< 0.001
Female Sex	1.542	0.344	2.741	0.012
Age 30 to 49	-0.600	-1.870	0.670	0.354
Age 50 or above	-1.634	-3.608	0.340	0.105
Youth Development Index	-0.047	-0.067	-0.027	0.000
Civic Engagement Index	-0.022	-0.037	-0.006	0.006
Financial Life Index	-0.033	-0.055	-0.012	0.002
Employed part-time but want full-time	-1.982	-4.192	0.227	0.079
Out of the workforce	-2.161	-3.591	-0.731	0.003
Completed secondary education	1.214	0.025	2.403	0.045
Needs more AT	1.475	-0.354	3.304	0.114
Age 50 or above * Out of the workforce	2.822	0.221	5.423	0.034
Capacity Score * Employed part-time but want full-time	-0.079	-0.170	0.012	0.088

In Laos, each additional point of capacity was associated with an average increase of 0.650 (95% CI: 0.624 to 0.676) in disability score in the minimum-parameter model (Table S3). Surprisingly, an age of 30 to 49 and age of 50 or above were negatively associated with disability.

The final stepwise model for Laos retained many demographic and environmental factors (Table 4). These factors improved the fit of the model compared to the minimum-parameter model (likelihood ratio test: *F* = 8.3107, *p* < 0.001). In the final model, the association between capacity and disability was slightly attenuated: each additional point of capacity was associated with an increase of 0.473 (95% CI: 0.395 to 0.551) in disability score. Even after further adjustments for demographic and environmental factors in the final model, an age of 30 to 49 and an age of 50 or above were negatively associated with disability in Laos. Being 30- to 49-years-old compared to 15- to 29-years-old was associated with a change in average disability score of -1.811 (95% CI: -2.947 to -0.674). Similarly, being 50 or above was associated with a change in average disability score of -2.690 (95% CI: -4.084 to -1.296) compared to being 15- to 29-years-old. An increase in the Community Basics Index of one point (on a 100-point scale) was associated with a change in disability score of -0.119 (95% CI: -0.159 to -0.079). Having AT & not needing more AT was associated with a -5.386 (95% CI: -8.613 to -2.159) point decrease in disability score compared to the baseline category of “not having AT & not needing AT.” Compared to the same baseline, needing more AT was associated with an increase in disability score of 1.475 (95% CI: 0.139 to 2.811). Being married was associated with an increase in disability score of 2.712 (95% CI: 0.315 to 5.109).

**Table 4:** Final linear model after stepwise addition of input variables for Laos. *R*^*2*^ = 0.515

	**Value**	**Confidence Interval**		***p*-value**
**Term**		**Lower 95%**	**Upper 95%**	
(Intercept)	27.127	23.211	31.043	< 0.001
Capacity Score	0.473	0.395	0.551	< 0.001
Female Sex	0.090	-0.849	1.029	0.850
Age 30 to 49	-1.811	-2.947	-0.674	0.002
Age 50 or above	-2.690	-4.084	-1.296	< 0.001
Community Basics Index	-0.119	-0.159	-0.079	< 0.001
Per Capita Income Quintiles	0.249	-0.368	0.866	0.429
Need more AT	1.475	0.139	2.811	0.031
Have AT and do not need more AT	-5.386	-8.613	-2.159	0.001
Married	2.712	0.315	5.109	0.027
Lives in a large city	0.609	-2.538	3.757	0.704
Civic Engagement Index	-0.015	-0.031	0.002	0.080
Employed full-time for an employer	1.110	-0.216	2.436	0.101
Financial Life Index	-0.013	-0.040	0.013	0.330
Social Life Index	0.014	-0.003	0.031	0.100
Capacity Score * Community Basics Index	0.002	0.001	0.003	< 0.001
Per Capita Income Quintiles * Have AT and do not need more AT	1.243	0.341	2.145	0.007
Per Capita Income Quintiles * Married	-1.320	-2.028	-0.612	< 0.001
Lives in large city * Financial Life Index	-0.066	-0.136	0.005	0.067

Four interactions were retained in the final stepwise model for Laos. While marriage was associated with a higher disability score, the interaction between marriage and per capita income quintile had a negative coefficient of -1.320 (95% CI: -2.028 to -0.612). The minimum-parameter and final stepwise models for Laos had *R*^*2*^ values of 0.494 and 0.515, indicating that additional adjustment increased the variance explained by 2.1%.

In Tajikistan, the capacity score was strongly associated with the disability score in the minimum-parameter model, with a coefficient of 0.799 (95% CI: 0.772 to 0.826) (Table S4). The final model for Tajikistan (Table 5) was a better fit to the data than the minimum-parameter model (likelihood ratio test: *F* = 27.294, *p* < 0.001). The association between capacity and disability was moderately attenuated in the final model, with each point increase in capacity score corresponding to an average increase in disability score of 0.776 (95% CI: 0.733 to 0.818). Sex was not significantly associated with disability in Tajikistan. An age of 50 or above was not associated with disability as an individual variable, but the interaction between capacity score and being age 50 or above had a strong association with a coefficient of 0.204 (95% CI: 0.147 to 0.261). An age of 30 to 49 was associated with a disability score that was 1.303 (95% CI: 0.296 to 2.311) higher than that of the average 15- to 29-year-old. The Community Basics Index was strongly negatively associated with disability score, with an average change in disability score of -0.076 (95% CI: -0.100 to -0.053) per index point. An increase in per capita income was associated with a reduction in disability score of -0.656 (95% CI: -0.977 to -0.334) for each income quintile. The Law-and-Order Score was negatively associated with disability score, with an average change in disability score of -0.058 (95% CI: -0.080 to -0.035) per index point. Needing more AT was associated with an increase in disability score of 2.078 (95% CI: 0.334 to 3.821) compared to the baseline category of not having AT & not needing AT. Being out of the workforce was associated with an increase in disability score of 1.454 (95% CI: 0.291 to 2.618) compared to the baseline category of being unemployed. There were also interactions between capacity score & living in a large city and capacity score & Civic Engagement Index, which had coefficients of -0.189 (95% CI: -0.268 to -0.110) and -0.001 (95% CI: -0.002 to -0.001) respectively. The minimum-parameter and final linear models for Tajikistan had *R*^*2*^ values of 0.604 and 0.641, indicating that including contextual factors increased the variance explained by 3.7%.

**Table 5:** Final linear model after stepwise addition of input variables for Tajikistan. *R*^*2*^ = 0.641

		**Confidence Interval**		
**Term**	**Value**	**Lower 95% CI**	**Upper 95% CI**	***p*-value**
(Intercept)	18.972	15.789	22.155	< 0.001
Capacity Score	0.776	0.733	0.818	< 0.001
Female Sex	0.036	-0.878	0.949	0.939
Age 30 to 49	1.303	0.296	2.311	0.011
Age 50 or above	-1.561	-3.454	0.332	0.106
Community Basics Index	-0.076	-0.100	-0.053	< 0.001
Lives in a large city	-0.884	-2.986	1.218	0.410
Law and Order Score	-0.058	-0.080	-0.035	< 0.001
Per Capita Income Quintiles	-0.656	-0.977	-0.334	< 0.001
Civic Engagement Index	-0.004	-0.023	0.015	0.665
Employed part-time and do not want full-time	-1.136	-2.888	0.617	0.204
Need more AT	2.078	0.334	3.821	0.020
Out of the workforce	1.454	0.291	2.618	0.014
Employed full-time for self	2.247	-0.124	4.618	0.063
Employed part-time and want full-time	1.269	-0.382	2.919	0.132
Capacity Score * Age 50 or above	0.204	0.147	0.261	< 0.001
Capacity Score * Lives in a large city	-0.189	-0.268	-0.110	< 0.001
Capacity Score * Civic Engagement Index	-0.001	-0.002	-0.001	< 0.001

### Sensitivity analyses

Excluding the 256 participants with missing data resulted in 2926 (97.5% of original) participants in India, 2352 (93.9%) participants in Laos, and 2970 (99%) participants in Tajikistan. Excluding participants with missing data made no significant difference to the results.

## Discussion

These results demonstrate that a variety of demographic, environmental, and capacity factors are associated with disability, with a unique set of key factors in each country. Capacity was the strongest predictor of disabilities in all three countries, which is in line with prior findings that health conditions are strong predictors of disability [1, 6, 10, 23]. On the other hand, some demographic and environmental factors were moderately associated with disability while controlling for the effects of capacity, and these associations varied substantially between countries. It is important to note that contextual factors may also be indirectly associated with disability through their mediating impact on health conditions (and therefore capacity), but these indirect associations were not assessed in the present study.

The random forest models explained about 60% of the variance in disability, which is similar to the percentages reported in previous studies using the MDS [6] and another disability survey [24]. The linear regression models captured most of the variance explained by the more complex random forest models, suggesting that the linear models were well-suited for these analyses. Most of the variance in disability score was explained by the minimum-parameter models that included only capacity score, sex, and age. The stepwise-constructed final models explained a small amount of additional variance compared to the minimum-parameter models. These results suggest that demographic and environmental factors have a modest yet significant association with disability across all three countries when controlling for the effects of capacity.

There were statistically significant interactions that were retained in the final linear regression models, indicating that demographic, environmental, and health factors may contribute to disability in a combinatorial fashion. To the best of the authors’ knowledge, such interactions have not been previously studied. These interactions provide valuable insight into disparities and potential avenues for intervention. For example, in Tajikistan, there was a strong negative interaction between capacity score and living in a large city. This interaction indicates that for people with more severe capacity limitations, living in a large city is associated with lower levels of disability. Thus, rural settings could be a focus of policy interventions to address disability in Tajikistan.

Regarding the strength of the associations between specific variables and disability across the three countries, sex was associated with disability only in India, where being female was associated with higher disability. This finding is in line with a previous study [25].

India had no significant associations between age and disability score aside from an interaction between being age 50 or above & being out of the workforce. This interaction was associated with greater disability. In Tajikistan, an age of 30 to 49 was associated with a higher disability, but an age of 50 or above alone was not significantly associated with disability. However, the interaction between capacity score and an age of 50 or above was a strong predictor of the disability score. Surprisingly, greater age was associated with lower disability scores for people in Laos, with a stronger reduction for people aged 50 or above compared to people aged 30 to 49. This result is unexpected because age is typically accompanied by an increase in the prevalence of chronic health conditions [26]. Three plausible explanations for the negative association between age and disability in Laos are 1) government programs that benefit the elderly, such as the National Policy for the Elderly and the Ministry of Labour and Social Welfare [27], 2) a strong societal and cultural respect for the elderly [28], and 3) a survivor bias in which people who live to old age tend to have lower levels of disability.

A higher per capita income quintile was associated with reduced disability in Tajikistan, while a higher Financial Life Index was associated with less disability in India. These results are logical given that financial stability is associated with reduced rates of disability [29]. In Laos, per capita income quintile interacted with two variables: have AT & do not need more AT, and marital status.

Being out of the workforce was the only employment variable that was associated with disability in any of the three countries. This is unexpected given that the type of employment (part-time vs full-time, self-employed vs for an employer) has been previously shown to be associated with disability [30, 31]. In Tajikistan, being out of the workforce was associated with a higher level of disability. Tajikistan had a high percentage of people out of the workforce, which may be due to lower economic participation by women in the Muslim-majority country [32]. On the other hand, being out of the workforce was associated with lower disability in India, but an interaction term between age 50 or above and being out of the workforce was positive. Thus, the association between being out of the workforce and disability is difficult to interpret in India.

People may experience disability differently in urban versus rural settings. Previous studies have reported disparities in the prevalence of health conditions and disability between urban and rural areas, with rural areas having worse outcomes due to poorer access to healthcare and lower levels of development [33, 34]. Living in a large city was only associated with disability in Tajikistan, in which an interaction between capacity score and living in a large city was associated with lower disability, as described above.

Public services and infrastructure are known to be important factors that affect quality of life for people with higher levels of disability [6]. The Community Basics Index (but not the Youth Development Index) was negatively associated with disability in Laos and Tajikistan. On the other hand, the Youth Development Index (but not the Community Basics Index) was negatively associated with disability in India. These findings suggest that investment in infrastructure and youth development programs could be an effective way to reduce disability in these settings.

Participation in society and service to others are known to be associated with disability [6]. In this study, the Civic Engagement Index was associated with lower levels of disability in India and Laos. In Tajikistan, the interaction between the Civic Engagement Index and capacity score was negatively associated with disability. These results are consistent with previous findings and suggest that creating more opportunities for participation in society and public service could reduce disability.

Using AT can significantly improve a person’s quality of life and everyday functioning [35]. In Laos and Tajikistan, people who needed more AT had higher levels of disability on average than people who did not use AT & did not need AT. These results suggest that AT provision could be a focus area for pilot interventions in Laos and Tajikistan.

Strengths of this study are the comprehensive, culturally-sensitive data collection protocols and the national representativeness of the samples. The MDS permitted accurate measurement of people’s capacity and disability levels, the key predictor and outcome variables, respectively. The wide array of demographic and environmental factors measured by the Gallup World Poll captured a fairly complete picture of each participant’s life.

The main limitation of this study is the cross-sectional design. Cross-sectional studies are susceptible to confounding by cohort effects, in which unique characteristics of a particular study population may distort the observed associations or reduce generalizability of the results. In addition, there is a potential for reverse causality in cross-sectional studies. Another limitation is that many variables were self-reported. Self-reporting may lead to measurement biases, such as recall bias, wherein participants give inaccurate responses to survey questions due to imperfect memories of the past. Despite these limitations, the correlations between the factors and disability can be used to guide future research and policymaking.

Taken together, these findings indicate that governments in resource-constrained settings should create public policies that address key demographic and environmental factors that are associated with disability in their specific local context. For instance, after identifying public infrastructure as a key factor, a government could make a commitment to develop a public transportation system that is accessible to all. Such policy interventions can often be cost-effective, yield great benefits for everyone in the society, and make progress towards fulfilling the CRPD and United Nations Sustainable Development Goals.

## Conclusion

The main finding of this study is that while health conditions (capacity) are the strongest determinant of disability, some demographic and environmental factors are associated with disability even while controlling for the effects of capacity. The contextual factors that were most consistently associated with disability were financial status, need for AT, basic community infrastructure & public services, and youth development. Notably, the associations between these factors and disability varied substantially across countries. Even factors that were expected to be universal correlates of disability, such as age, had different associations with disability in each country. The cross-country variation in demographic and environmental factors associated with disability highlights the importance of understanding local context when designing policy interventions for disability.

These findings also demonstrate the utility of the B-MDS for measuring capacity and disability according to the ICF definitions. The joint implementation with Gallup yielded a rich dataset of demographic and environmental factors in a highly efficient, culturally-sensitive, and standardized manner.

The heterogeneity of the relationships between the factors and disability highlights the importance of expanding the MDS to other countries around the world. Additional research is necessary to assess disability needs worldwide and to compare key demographic and environmental factors across settings. A greater understanding of disability is necessary to improve global well-being and reduce disparities, ensuring that no one is left behind.

## Supplementary Information

**Additional file 1: Methods 1. **Brief MDS Questionnaire.

**Additional file 2: Methods 2.** Excluded Gallup Indices. **Table S1.** Variableimportance measures from random forest regression models for disability score. **Figure S1. **Examples of feature contributionplots generated from the trained random forest model for Laos. **Figure S2. **A combined feature contribution plotof capacity score and community basics index reveals a significant interactionbetween the variables. **Table S2. **Minimum-parameter linear regression model for disabilityscore in India. **Table S3. **Minimum-parameter linear regression model fordisability score in Laos. **Table S4. **Minimum-parameter linear regression model fordisability score in Tajikistan.

## Data Availability

Data is available from the corresponding author upon request.

## Abbreviations

AIC: Akaike information criterion
AT: Assistive technology
B-MDS: Brief Model Disability Survey
CI: Confidence interval
CRPD: Convention on the Rights of Persons with Disabilities
ICF: International Classification of Functioning, Disability, and Health
IQR: Inter-quartile range
LMIC: Low and middle-income country
MDS: Model Disability Survey
SD: Standard deviation
VIM: Variable importance measure
WHO: World Health Organization
