# Supplementary material for: Demographic and environmental factors associated with disability in India, Laos, and Tajikistan: a population-based cross-sectional study
Source: BMC Public Health. 2022 Mar 29;22:607. doi: 10.1186/s12889-022-12846-1 (PMC8962048; doi:10.1186/s12889-022-12846-1)
Supplement: Supplementary file 1 — Additional file 1: Methods 1. Brief MDS Questionnaire. [file 12889_2022_12846_MOESM1_ESM.docx]

**[[Country] 2018 – WHO section World Poll F2F CAPI](https://qbank.gallup.com/QBank5/SurveyBuilder.aspx?QNAIREID=18130&METHID=31&LANGID=1)**

2018

**______________________________________________________________________**

**SA.** [WP5]

COUNTRY:

| [Country] |  |
| --- | --- |

**SC5.** [WP17638]

***(READ:)*** We are now beginning a new section of the survey, and I am going to ask you again for your consent to participate in this section. This next section will take approximately 12 minutes and is about how people live their lives in **[Country]**. We are trying to understand the kinds of problems adults experience in their daily life in your community, such as with mobility or doing household tasks. These problems might arise because of a person's health or other barriers, like inaccessible transportation or discriminatory attitudes of others. We also want to identify unmet needs that could relieve these problems. If you do not understand anything or if you have any questions, please let me know.

As mentioned before, we are talking to randomly selected people like you.

During this part of the survey, I will read questions aloud to you, ask you to respond to them and record your response in an electronic system, as we have for other sections.

Please remember that all information you tell us is completely confidential and will only be used for research purposes. All information will be kept private. Any information about you will have a number on it instead of your name and will not be shared with anyone outside of the research team.

We are asking you to share with us some information about problems. You may feel that some of this information is sensitive or personal. You do not have to answer any question or take part in this section of the survey if you don't wish to do so. That is fine. You do not have to give us any reason for not responding to any question, or for refusing to take part in this section of the survey.

We will compensate you for completing this section of the survey with **21 somonis**.

You do not have to participate if you do not wish to do so, and choosing to participate will not affect any benefits or services you may receive in any way. You may choose to stop participating and skip this section of the survey at any time that you wish.

Your participation may inform policies or projects that aim to improve the lives of people in **[Country]**. We will publish results from this section for the population, and will share our findings online and directly with local civil society organizations, so that you may learn about it.

If you have any questions you can ask me now or you can contact the responsible colleague at Gallup, Mr. Andrew Rzepa, andrew_rzepa@gallup.co.uk, or the responsible colleague at the World Health Organization, Ms. Lindsay Lee, leel@who.int.

Before I start this section, I will need you to confirm that I have read the information about the questions; that you have had the opportunity to ask questions about them, and that any questions you have asked have been answered to your satisfaction. Is this true? ***(Interviewer: If respondent says "no", ask what questions they have and reread informed consent, if necessary)***

**H0.** [WP20415]

Do you consent voluntarily to continue with this section of the survey?

|  | **CIRCLE ONE RESPONSE:** | **ROUTE:** |
| --- | --- | --- |
| Yes | 1 | **(Continue)** |
| No | 2 | **(Thank and Terminate)** |
| (DK) | 98 |  |
| (Refused) | 99 |  |

**H1.** Now, I am going to ask you some general questions about your environment and your social relationships. I want you to answer the following questions on a scale from 1 to 5, where 1 means very easy and 5 means very hard. ***(Read H1A-H1G)*** ***(Interviewer: Use show card H1 and read categories, if necessary.)***

|  | | **Very easy** |  |  |  | **Very hard** | **(Not applicable)** | **(DK)** | **(Refused)** |
| --- | --- | --- | --- | --- | --- | --- | --- | --- | --- |
| **H1A.** [WP20416] | How easy or hard is it for you to use places where you socialize and engage in community activities? | 1 | 2 | 3 | 4 | 5 | 97 | 98 | 99 |
| **H1B.** [WP20417] | How easy or hard is it for you to use shops, banks, and the post office in your neighborhood? | 1 | 2 | 3 | 4 | 5 | 97 | 98 | 99 |
| **H1C.** [WP20418] | How easy or hard is it for you to use transportation you need or want to use? | 1 | 2 | 3 | 4 | 5 | 97 | 98 | 99 |
| **H1D.** [WP20419] | How easy or hard is it for you to live in your dwelling, including using the toilet? | 1 | 2 | 3 | 4 | 5 |  | 98 | 99 |
| **H1E.** [WP20420] | If you needed help, how easy or hard would it be for you to get help from a close family member [***(Programmer: If code 2 or 8 in Q6C/WP1223, ADD:)*** including your partner]? | 1 | 2 | 3 | 4 | 5 | 97 | 98 | 99 |
| **H1F.** [WP20421] | If you needed help, how easy or hard would it be for you to get help from friends or co-workers? | 1 | 2 | 3 | 4 | 5 | 97 | 98 | 99 |
| **H1G.** [WP20422] | If you needed help, how easy or hard would it be for you to get help from neighbors? | 1 | 2 | 3 | 4 | 5 | 97 | 98 | 99 |

***(Programmer: If respondent says "don't know" [code 98] or "refused" [code 99] to three items in a row, Continue;
Otherwise, Skip to Text before H2/WP20423)***

**H1H.** [H1H]

You do not have to answer any question or take part in this section of the survey if you don't wish to do so. We will compensate you for completing this section of the survey with **21 somonis**. Do you wish to continue with this section?

|  | **CIRCLE ONE RESPONSE:** | **ROUTE:** |
| --- | --- | --- |
| Yes | 1 | **(Continue)** |
| No | 2 | **(Thank and Terminate)** |
| (DK) | 98 |  |
| (Refused) | 99 |  |

**H2.** Please answer the following two questions on a scale from 1 to 5, where 1 means not at all and 5 means completely. ***(Read H2A-H2B)*** ***(Interviewer: Use show card H2 and read categories, if necessary.)***

|  | | **Not at all** |  |  |  | **Completely** | **(DK)** | **(Refused)** |
| --- | --- | --- | --- | --- | --- | --- | --- | --- |
| **H2A.** [WP20423] | To what extent do you make your own choices about your day-to-day life? For example, where to go, what to do, and what to eat. | 1 | 2 | 3 | 4 | 5 | 98 | 99 |
| **H2B.** [WP20424] | To what extent do you feel that other people respect you, value you as a person, and listen to what you have to say? | 1 | 2 | 3 | 4 | 5 | 98 | 99 |

**H3.** The next questions ask about problems you may experience in your life. By problems, I mean not getting things done in the way you want to or not getting things done at all. These problems may arise because of your health, the environment in which you live, or because of the attitudes or behaviors of others. Please think about the last 30 days, taking both good and bad days into account. For each question, please tell me how much of a problem it is for you on a scale from 1 to 5, where 1 means "no problem" and 5 means "extreme problem." Please take into account your health, people who help you, and any assistive products you use or medication you take. ***(Read H3A-H3L)*** ***(Interviewer: Use show card H3 and read categories, if necessary.)***

|  | | **No problem** |  |  |  | **Extreme problem** | **(Not applicable)** | **(DK)** | **(Refused)** |
| --- | --- | --- | --- | --- | --- | --- | --- | --- | --- |
| **H3A.** [WP20425] | How much of a problem is walking a kilometer for you? | 1 | 2 | 3 | 4 | 5 |  | 98 | 99 |
| **H3B.** [WP20426] | How much of a problem is getting where you want to go for you? | 1 | 2 | 3 | 4 | 5 |  | 98 | 99 |
| **H3C.** [WP20427] | How much of a problem is being clean and dressed? | 1 | 2 | 3 | 4 | 5 |  | 98 | 99 |
| **H3D.** [WP20428] | How much of a problem is using the toilet? | 1 | 2 | 3 | 4 | 5 |  | 98 | 99 |
| **H3E.** [WP20429] | How much of a problem is looking after your health, eating well, exercising, or taking your medicines? | 1 | 2 | 3 | 4 | 5 |  | 98 | 99 |
| **H3F.** [WP20430] | How much of a problem is feeling tired and not having enough energy? | 1 | 2 | 3 | 4 | 5 |  | 98 | 99 |
| **H3G.** [WP20431] | How much of a problem is coping with all the things you have to do? | 1 | 2 | 3 | 4 | 5 |  | 98 | 99 |
| **H3H.** [WP20432] | How much of a problem is remembering to do the important things in your day-to-day life? | 1 | 2 | 3 | 4 | 5 |  | 98 | 99 |
| **H3I.** [WP20433] | How much of a problem do you have with getting your household tasks done? | 1 | 2 | 3 | 4 | 5 |  | 98 | 99 |
| **H3J.** [WP20434] | How much of a problem do you have with joining community activities, such as festivities, religious or other activities? | 1 | 2 | 3 | 4 | 5 |  | 98 | 99 |
| **H3K.** [WP20435] | How much of a problem is using public or private transportation? | 1 | 2 | 3 | 4 | 5 | 97 | 98 | 99 |
| **H3L.** [WP20436] | How much of a problem is it to get things done as required at work or school? ***(Interviewer: If respondent says they are not working or not in school, select "Not applicable")*** | 1 | 2 | 3 | 4 | 5 | 97 | 98 | 99 |

**H4.** [WP20437]

I have asked you many questions about problems you may experience in your life. The next questions ask about difficulties you may have doing certain activities only because of your HEALTH. Please think about the last 30 days, taking both good and bad days into account. I will start with a question about your overall health, including your physical and mental health. In general, how would you rate your health today? Very good, good, neither good nor poor, poor, or very poor? ***(Interviewer: Use show card H4 and read categories, if necessary.)***

|  | **CIRCLE ONE RESPONSE:** |
| --- | --- |
| Very good | 1 |
| Good | 2 |
| Neither good nor poor | 3 |
| Poor | 4 |
| Very poor | 5 |
| (DK) | 98 |
| (Refused) | 99 |

**H5.** The next questions ask about difficulties you may have doing certain activities because of a HEALTH PROBLEM. I want you to answer the questions on a scale from 1 to 5, where 1 means "no difficulty" and 5 means "extreme difficulty or you cannot do the activity at all." Please answer these questions WITHOUT taking into account any help you may receive. ***(Interviewer: Read H5A-H5K)*** ***(Use show card H5 and read categories, if necessary.)***

|  | | **No difficulty** |  |  |  | **Extreme difficulty or you cannot do the activity at all** | **(DK)** | **(Refused)** |
| --- | --- | --- | --- | --- | --- | --- | --- | --- |
| **H5A.** [WP20438] | How much difficulty do you have seeing things at a distance without glasses? | 1 | 2 | 3 | 4 | 5 | 98 | 99 |
| **H5B.** [WP20439] | How much difficulty do you have hearing without hearing aids? | 1 | 2 | 3 | 4 | 5 | 98 | 99 |
| **H5C.** [WP20440] | How much difficulty do you have walking or climbing steps? | 1 | 2 | 3 | 4 | 5 | 98 | 99 |
| **H5D.** [WP20441] | How much difficulty do you have remembering or concentrating? | 1 | 2 | 3 | 4 | 5 | 98 | 99 |
| **H5E.** [WP20442] | How much difficulty do you have washing all over or dressing? | 1 | 2 | 3 | 4 | 5 | 98 | 99 |
| **H5F.** [WP20443] | How much difficulty do you have sleeping because of your health? | 1 | 2 | 3 | 4 | 5 | 98 | 99 |
| **H5G.** [WP20444] | How much difficulty do you have doing household tasks because of your health? | 1 | 2 | 3 | 4 | 5 | 98 | 99 |
| **H5H.** [WP20445] | Because of your health, how much difficulty do you have with joining community activities, such as festivities, religious or other activities? | 1 | 2 | 3 | 4 | 5 | 98 | 99 |
| **H5I.** [WP20446] | How much difficulty do you have with feeling sad, low, worried or anxious because of your health? | 1 | 2 | 3 | 4 | 5 | 98 | 99 |
| **H5J.** [WP20447] | Because of your health, how much difficulty do you have getting along with people who are close to you, including your family and friends? | 1 | 2 | 3 | 4 | 5 | 98 | 99 |
| **H5K.** [WP20448] | How much difficulty do you have with bodily aches or pain? | 1 | 2 | 3 | 4 | 5 | 98 | 99 |

**H6.** Do you currently have any of these diseases or health problems? ***(Interviewer: Read H6A-H6P)***

|  | | **Yes** | **No** | **(DK)** | **(Refused)** |
| --- | --- | --- | --- | --- | --- |
| **H6A.** [WP20449] | Vision loss | 1 | 2 | 98 | 99 |
| **H6B.** [WP20450] | Hearing loss - that is, the partial or total inability to hear | 1 | 2 | 98 | 99 |
| **H6C.** [WP20451] | High blood pressure/hypertension | 1 | 2 | 98 | 99 |
| **H6D.** [WP20452] | Heart disease, coronary disease, heart attack | 1 | 2 | 98 | 99 |
| **H6E.** [WP20453] | Stroke - that is, damage to the brain caused by disruption of the blood supply. A stroke can cause the permanent or temporary inability to move, usually down one side of the body, and loss of speech. | 1 | 2 | 98 | 99 |
| **H6F.** [WP20454] | Diabetes - a long-term condition where a person has problems producing insulin. | 1 | 2 | 98 | 99 |
| **H6G.** [WP20455] | Arthritis or arthrosis - a disease of joints, which may cause swelling, stiffness, redness, heat and/or pain in fingers/wrists, knees, hips, or lower back. | 1 | 2 | 98 | 99 |
| **H6H.** [WP20456] | Chronic bronchitis or emphysema [***(If necessary, Read:)*** Lung diseases that cause frequent problems with breathing, usually including shortness of breath and coughing up sputum. This does not include asthma.] | 1 | 2 | 98 | 99 |
| **H6I.** [WP20457] | Asthma or allergic respiratory disease; that is, reoccurring attacks of breathlessness and wheezing. | 1 | 2 | 98 | 99 |
| **H6J.** [WP20458] | Back pain | 1 | 2 | 98 | 99 |
| **H6K.** [WP20459] | Depression - a persistent depressed mood or loss of interest in activities affecting one's daily life over a period of time. | 1 | 2 | 98 | 99 |
| **H6L.** [WP20460] | Anxiety - a persistent feeling of fear, worry, and uneasiness. | 1 | 2 | 98 | 99 |
| **H6M.** [WP20461] | Amputation - the removal of a body extremity by trauma or surgery. | 1 | 2 | 98 | 99 |
| **H6N.** [WP20462] | Trauma from an accident or event that resulted in bodily injury. | 1 | 2 | 98 | 99 |
| **H6O.** [WP20463] | Tinnitus - ringing, roaring, or buzzing in your ears that lasts for five minutes or longer. | 1 | 2 | 98 | 99 |
| **H6P.** [WP20464] | Do you have any other diseases or health problems? | 1 | 2 | 98 | 99 |

**H7.** [WP20465]

Do you have someone to assist you with your day-to-day activities at home or outside of the home?

|  | **CIRCLE ONE RESPONSE:** | **ROUTE:** |
| --- | --- | --- |
| Yes | 1 | **(Continue)** |
| No | 2 | **(Skip to H9/WP20467)** |
| (DK) | 98 |  |
| (Refused) | 99 |  |

**H8.** [WP20466]

Do you think you need additional assistance with your day-to-day activities at home or outside of the home?

|  | **CIRCLE ONE RESPONSE:** | **ROUTE:** |
| --- | --- | --- |
| Yes | 1 | **(Skip to H10/Text before WP20468)** |
| No | 2 |  |
| (DK) | 98 |  |
| (Refused) | 99 |  |

**H9.** [WP20467]

Do you think you need someone to assist you with your day-to-day activities at home or outside of the home?

|  | **CIRCLE ONE RESPONSE:** |
| --- | --- |
| Yes | 1 |
| No | 2 |
| (DK) | 98 |
| (Refused) | 99 |

**H10.** Do you currently use any of these assistive products? ***(Interviewer: Read H10A-H10Q and use show cards H10A-H10Q)***

|  | | **Yes** | **No** | **(DK)** | **(Refused)** |
| --- | --- | --- | --- | --- | --- |
| **H10A.** [WP20468] | ***(Interviewer: Show card H10A)*** Canes or walking sticks | 1 | 2 | 98 | 99 |
| **H10B.** [WP20469] | ***(Interviewer: Show card H10B)*** Crutches | 1 | 2 | 98 | 99 |
| **H10C.** [WP20470] | ***(Interviewer: Show card H10C)*** Orthosis or brace - a fitted medical device used on a foot, leg, arm, hand, or the spine | 1 | 2 | 98 | 99 |
| **H10D.** [WP20471] | ***(Interviewer: Show card H10D)*** Tricycle | 1 | 2 | 98 | 99 |
| **H10E.** [WP20472] | ***(Interviewer: Show card H10E)*** Pressure relief cushions | 1 | 2 | 98 | 99 |
| **H10F.** [WP20473] | ***(Interviewer: Show card H10F)*** Prosthesis/artificial leg | 1 | 2 | 98 | 99 |
| **H10G.** [WP20474] | ***(Interviewer: Show card H10G)*** Walking frame or rollator | 1 | 2 | 98 | 99 |
| **H10H.** [WP20475] | ***(Interviewer: Show card H10H)*** Chair for the shower, bath, or toilet | 1 | 2 | 98 | 99 |
| **H10I.** [WP20476] | ***(Interviewer: Show card H10I)*** Manual wheelchair | 1 | 2 | 98 | 99 |
| **H10J.** [WP20477] | ***(Interviewer: Show card H10J)*** Electric wheelchair | 1 | 2 | 98 | 99 |
| **H10K.** [WP20478] | ***(Interviewer: Show card H10K)*** Incontinence products | 1 | 2 | 98 | 99 |
| **H10L.** [WP20479] | ***(Interviewer: Show card H10L)*** Magnifier | 1 | 2 | 98 | 99 |
| **H10M.** [WP20480] | ***(Interviewer: Show card H10M)*** Spectacles/glasses | 1 | 2 | 98 | 99 |
| **H10N.** [WP20481] | ***(Interviewer: Show card H10N)*** A white cane used by people with partial or complete blindness | 1 | 2 | 98 | 99 |
| **H10O.** [WP20482] | ***(Interviewer: Show card H10O)*** Hearing aids | 1 | 2 | 98 | 99 |
| **H10P.** [WP20483] | ***(Interviewer: Show card H10P)*** Communication boards, books, or cards | 1 | 2 | 98 | 99 |
| **H10Q.** [WP20484] | ***(Interviewer: Show card H10Q)*** Products for memory support, for example, a pill organizer | 1 | 2 | 98 | 99 |

***(If "yes" [code 1] to ANY in H10A/WP20468 - H10Q/WP20484, Continue;
Otherwise, Skip to H16/Text before WP20520)***

**H11.** [WP20485]

To what extent do your assistive products make your life easier? Completely, very much, moderately, a little, or not at all? ***(Interviewer: Use show card H11)***

|  | **CIRCLE ONE RESPONSE:** |
| --- | --- |
| Completely | 5 |
| Very much | 4 |
| Moderately | 3 |
| A little | 2 |
| Not at all | 1 |
| (DK) | 98 |
| (Refused) | 99 |

**H12.** [WP20486]

Do you face any problems using any of your assistive products?

|  | **CIRCLE ONE RESPONSE:** | **ROUTE:** |
| --- | --- | --- |
| Yes | 1 | **(Continue)** |
| No | 2 | **(Skip to H14/WP20502)** |
| (DK) | 98 |  |
| (Refused) | 99 |  |

**H13.** Which of the following problems do you currently face with any of your assistive products? ***(Interviewer: Read H13A-H13O)***

|  | | **Yes** | **No** | **(DK)** | **(Refused)** |
| --- | --- | --- | --- | --- | --- |
| **H13A.** [WP20487] | Assistive product is not the right size | 1 | 2 | 98 | 99 |
| **H13B.** [WP20488] | Assistive product is not suitable for your home or surroundings | 1 | 2 | 98 | 99 |
| **H13C.** [WP20489] | Assistive product is not safe | 1 | 2 | 98 | 99 |
| **H13D.** [WP20490] | Assistive product is not helpful | 1 | 2 | 98 | 99 |
| **H13E.** [WP20491] | Assistive product is not comfortable | 1 | 2 | 98 | 99 |
| **H13F.** [WP20492] | Assistive product is a self-made, temporary solution | 1 | 2 | 98 | 99 |
| **H13G.** [WP20493] | Assistive product needs maintenance or replacements that are not available | 1 | 2 | 98 | 99 |
| **H13H.** [WP20494] | Assistive product is broken | 1 | 2 | 98 | 99 |
| **H13I.** [WP20495] | No one showed you how to use the assistive product | 1 | 2 | 98 | 99 |
| **H13J.** [WP20496] | Assistive product is complicated to use | 1 | 2 | 98 | 99 |
| **H13K.** [WP20497] | You need help from another person to use the assistive product | 1 | 2 | 98 | 99 |
| **H13L.** [WP20498] | You share the assistive product with other people | 1 | 2 | 98 | 99 |
| **H13M.** [WP20499] | You find your assistive product embarrassing | 1 | 2 | 98 | 99 |
| **H13N.** [WP20500] | You feel like people treat you differently when you use your assistive product | 1 | 2 | 98 | 99 |
| **H13O.** [WP20501] | Do you have any other problems using your assistive products? | 1 | 2 | 98 | 99 |

**H14.** [WP20502]

In addition to what you use, do you think you need any other assistive products?

|  | **CIRCLE ONE RESPONSE:** | **ROUTE:** |
| --- | --- | --- |
| Yes | 1 | **(Continue)** |
| No | 2 | **(Skip to SC5B/WP17772)** |
| (DK) | 98 |  |
| (Refused) | 99 |  |

**H15.** Which of the following assistive products do you think you need that you do not already have? ***(Programmer: Display items below if code 2, 98, or 99 in H10A-H10Q) (Interviewer: Read items and use show cards H15A-H15Q, as appropriate)***

|  | | **Yes** | **No** | **(DK)** | **(Refused)** |
| --- | --- | --- | --- | --- | --- |
| **H15A.** [WP20503] | ***(Interviewer: Show card H15A)*** Canes or walking sticks | 1 | 2 | 98 | 99 |
| **H15B.** [WP20504] | ***(Interviewer: Show card H15B)*** Crutches | 1 | 2 | 98 | 99 |
| **H15C.** [WP20505] | ***(Interviewer: Show card H15C)*** Orthosis or brace - a fitted medical device used on a foot, leg, arm, hand, or the spine | 1 | 2 | 98 | 99 |
| **H15D.** [WP20506] | ***(Interviewer: Show card H15D)*** Tricycle | 1 | 2 | 98 | 99 |
| **H15E.** [WP20507] | ***(Interviewer: Show card H15E)*** Pressure relief cushions | 1 | 2 | 98 | 99 |
| **H15F.** [WP20508] | ***(Interviewer: Show card H15F)*** Prosthesis/artificial leg | 1 | 2 | 98 | 99 |
| **H15G.** [WP20509] | ***(Interviewer: Show card H15G)*** Walking frame or rollator | 1 | 2 | 98 | 99 |
| **H15H.** [WP20510] | ***(Interviewer: Show card H15H)*** Chair for the shower, bath, or toilet | 1 | 2 | 98 | 99 |
| **H15I.** [WP20511] | ***(Interviewer: Show card H15I)*** Manual wheelchair | 1 | 2 | 98 | 99 |
| **H15J.** [WP20512] | ***(Interviewer: Show card H15J)*** Electric wheelchair | 1 | 2 | 98 | 99 |
| **H15K.** [WP20513] | ***(Interviewer: Show card H15K)*** Incontinence products | 1 | 2 | 98 | 99 |
| **H15L.** [WP20514] | ***(Interviewer: Show card H15L)*** Magnifier | 1 | 2 | 98 | 99 |
| **H15M.** [WP20515] | ***(Interviewer: Show card H15M)*** Spectacles/glasses | 1 | 2 | 98 | 99 |
| **H15N.** [WP20516] | ***(Interviewer: Show card H15N)*** A white cane used by people with partial or complete blindness | 1 | 2 | 98 | 99 |
| **H15O.** [WP20517] | ***(Interviewer: Show card H15O)*** Hearing aids | 1 | 2 | 98 | 99 |
| **H15P.** [WP20518] | ***(Interviewer: Show card H15P)*** Communication boards, books, or cards | 1 | 2 | 98 | 99 |
| **H15Q.** [WP20519] | ***(Interviewer: Show card H15Q)*** Products for memory support, for example, a pill organizer | 1 | 2 | 98 | 99 |

***(All in H15Q/WP20519, Skip to SC5B/WP17772)***

**H16.** You told me you do not use assistive products. Do you think you need any of the following assistive products? ***(Interviewer: Read H16A-H16Q and use show cards H15A-H15Q)***

|  | | **Yes** | **No** | **(DK)** | **(Refused)** |
| --- | --- | --- | --- | --- | --- |
| **H16A.** [WP20520] | ***(Interviewer: Show card H15A)*** Canes or walking sticks | 1 | 2 | 98 | 99 |
| **H16B.** [WP20521] | ***(Interviewer: Show card H15B)*** Crutches | 1 | 2 | 98 | 99 |
| **H16C.** [WP20522] | ***(Interviewer: Show card H15C)*** Orthosis or brace - a fitted medical device used on a foot, leg, arm, hand, or the spine | 1 | 2 | 98 | 99 |
| **H16D.** [WP20523] | ***(Interviewer: Show card H15D)*** Tricycle | 1 | 2 | 98 | 99 |
| **H16E.** [WP20524] | ***(Interviewer: Show card H15E)*** Pressure relief cushions | 1 | 2 | 98 | 99 |
| **H16F.** [WP20525] | ***(Interviewer: Show card H15F)*** Prosthesis/artificial leg | 1 | 2 | 98 | 99 |
| **H16G.** [WP20526] | ***(Interviewer: Show card H15G)*** Walking frame or rollator | 1 | 2 | 98 | 99 |
| **H16H.** [WP20527] | ***(Interviewer: Show card H15H)*** Chair for the shower, bath, or toilet | 1 | 2 | 98 | 99 |
| **H16I.** [WP20528] | ***(Interviewer: Show card H15I)*** Manual wheelchair | 1 | 2 | 98 | 99 |
| **H16J.** [WP20529] | ***(Interviewer: Show card H15J)*** Electric wheelchair | 1 | 2 | 98 | 99 |
| **H16K.** [WP20530] | ***(Interviewer: Show card H15K)*** Incontinence products | 1 | 2 | 98 | 99 |
| **H16L.** [WP20531] | ***(Interviewer: Show card H15L)*** Magnifier | 1 | 2 | 98 | 99 |
| **H16M.** [WP20532] | ***(Interviewer: Show card H15M)*** Spectacles/glasses | 1 | 2 | 98 | 99 |
| **H16N.** [WP20533] | ***(Interviewer: Show card H15N)*** A white cane used by people with partial or complete blindness | 1 | 2 | 98 | 99 |
| **H16O.** [WP20534] | ***(Interviewer: Show card H15O)*** Hearing aids | 1 | 2 | 98 | 99 |
| **H16P.** [WP20535] | ***(Interviewer: Show card H15P)*** Communication boards, books, or cards | 1 | 2 | 98 | 99 |
| **H16Q.** [WP20536] | ***(Interviewer: Show card H15Q)*** Products for memory support, for example, a pill organizer | 1 | 2 | 98 | 99 |

***(If "yes" [code 1] to ANY in H16A/WP20520 - H16Q/WP20536, Continue;
Otherwise, Skip to SC5B/WP17772)***

**H17.** Please tell me whether each of the following is a reason why you do not have the assistive products you need. ***(Interviewer: Read H17A-H17R)***

|  | | **Yes** | **No** | **(DK)** | **(Refused)** |
| --- | --- | --- | --- | --- | --- |
| **H17A.** [WP20537] | You did not know about the assistive product | 1 | 2 | 98 | 99 |
| **H17B.** [WP20538] | Assistive product is not available in your area | 1 | 2 | 98 | 99 |
| **H17C.** [WP20539] | You cannot afford the cost of the assistive product | 1 | 2 | 98 | 99 |
| **H17D.** [WP20540] | Available assistive products are not suitable for your home or surroundings | 1 | 2 | 98 | 99 |
| **H17E.** [WP20541] | Available assistive products are not safe | 1 | 2 | 98 | 99 |
| **H17F.** [WP20542] | Available assistive products are not helpful | 1 | 2 | 98 | 99 |
| **H17G.** [WP20543] | Available assistive products are not comfortable | 1 | 2 | 98 | 99 |
| **H17H.** [WP20544] | Available assistive products are too complicated to use | 1 | 2 | 98 | 99 |
| **H17I.** [WP20545] | You did not know where to go to get the assistive product | 1 | 2 | 98 | 99 |
| **H17J.** [WP20546] | You did not get the approval you needed to get the assistive product | 1 | 2 | 98 | 99 |
| **H17K.** [WP20547] | You thought you would not be eligible to get the assistive product | 1 | 2 | 98 | 99 |
| **H17L.** [WP20548] | No one is available to show you how to use the assistive product | 1 | 2 | 98 | 99 |
| **H17M.** [WP20549] | Suitable transportation is not available to get the assistive product | 1 | 2 | 98 | 99 |
| **H17N.** [WP20550] | You need assistance to use the assistive product, but assistance is not available | 1 | 2 | 98 | 99 |
| **H17O.** [WP20551] | You would feel embarrassed if you had the assistive product | 1 | 2 | 98 | 99 |
| **H17P.** [WP20552] | People would treat you differently if you had the assistive product | 1 | 2 | 98 | 99 |
| **H17Q.** [WP20553] | Your family does not want you to use the assistive product | 1 | 2 | 98 | 99 |
| **H17R.** [WP20554] | Are there any other reasons why you do not have the assistance products you need? | 1 | 2 | 98 | 99 |
