# Supplementary material for: Demographic and environmental factors associated with disability in India, Laos, and Tajikistan: a population-based cross-sectional study
Source: BMC Public Health. 2022 Mar 29;22:607. doi: 10.1186/s12889-022-12846-1 (PMC8962048; doi:10.1186/s12889-022-12846-1)
Supplement: Supplementary file 2 — Additional file 2: Methods 2. Excluded Gallup Indices. Table S1. Variableimportance measures from random forest regression models for disability score. Figure S1. Examples of feature contributionplots generated from the trained random forest model for Laos. Figure S2. A combined feature contribution plotof capacity score and community basics index reveals a significant interactionbetween the variables. Table S2. Minimum-parameter linear regression model for disabilityscore in India. Table S3. Minimum-parameter linear regression model fordisability score in Laos. Table S4. Minimum-parameter linear regression model fordisability score in Tajikistan. [file 12889_2022_12846_MOESM2_ESM.docx]

**Supplementary Methods 2: Excluded Gallup Indices**

The Communications Use Index, Communications Index, Job Climate Index, Economic Confidence Index, and Local Economic Confidence Index were excluded from the analyses due to redundancy with other Gallup Indices. The Life Evaluation Index, Positive Experience Index, Negative Experience Index, Daily Experience Index, and Personal Health Index were excluded from analyses due to suspected collinearity with disability score. The Corruption Index, National Institutions Index, Diversity Index, and Community Attachment Index were excluded from analyses due to a lack of data in one or more of the countries. In lieu of using the Law-and-Order Index from Gallup, a Law-and-Order score was calculated by omitting a recently added question about mugging/assault because this question had substantial missing data.

**Table S1:** **Variable importance measures from random forest regression models for disability score.** The variables are ordered by decreasing order of VIM in India. The VIM is equal to the percentage increase in mean squared error when the variable values are randomly shuffled.

| **Variable** | **India**  (R^2^ = 0.600) | | **Laos**  (R^2^ = 0.511) | | **Tajikistan**  (R^2^ = 0.669) | |
| --- | --- | --- | --- | --- | --- | --- |
|  | **VIM** | **Rank** | **VIM** | **Rank** | **VIM** | **Rank** |
| Capacity Score | 532.4 | 1 | 248.1 | 1 | 411.0 | 1 |
| Community Basics Index | 22.67 | 2 | 4.25 | 3 | 9.58 | 3 |
| Youth Development Index | 18.97 | 3 | 2.31 | 7 | 0.79 | 21 |
| Financial Life Index | 12.69 | 4 | 1.98 | 8 | 9.30 | 4 |
| Need more assistive technology | 9.54 | 5 | 1.72 | 10 | 4.42 | 10 |
| Have assistive technology and do not need more | 9.32 | 6 | 0.84 | 15 | 9.26 | 5 |
| Law and Order Score | 7.82 | 7 | 0.27 | 21 | 2.39 | 15 |
| Per Capita Income Quintiles | 6.80 | 8 | 4.66 | 2 | 7.23 | 6 |
| Age 50 or above | 6.28 | 9 | 2.92 | 5 | 15.41 | 2 |
| Communications Access Index | 6.11 | 10 | 2.31 | 6 | 5.23 | 8 |
| Civic Engagement Index | 5.79 | 11 | 1.90 | 9 | 1.98 | 17 |
| Social Life Index | 5.29 | 12 | 1.00 | 13 | 2.98 | 12 |
| Food and Shelter Index | 3.90 | 13 | 3.67 | 4 | 5.88 | 7 |
| Sex | 3.04 | 14 | 0.36 | 20 | 2.82 | 13 |
| Employed full-time for an employer | 2.73 | 15 | 1.58 | 11 | 1.54 | 18 |
| Age 30 to 49 | 2.71 | 16 | 0.71 | 17 | 2.47 | 14 |
| Completed secondary education | 2.57 | 17 | 1.58 | 12 | 1.26 | 19 |
| Out of the workforce | 2.43 | 18 | -0.10 | 24 | 3.35 | 11 |
| Married | 2.19 | 19 | 0.60 | 19 | 1.17 | 20 |
| Employed part-time and want full-time | 2.02 | 20 | 0.84 | 14 | 0.49 | 22 |
| Employed full-time for self | 0.84 | 21 | 0.83 | 16 | 0.14 | 23 |
| Lives in a large city | 0.74 | 22 | -0.01 | 23 | 4.83 | 9 |
| Completed tertiary education | 0.59 | 23 | 0.68 | 18 | 2.33 | 16 |
| Employed part-time and do not want full-time | -0.51 | 24 | 0.14 | 22 | -0.01 | 24 |


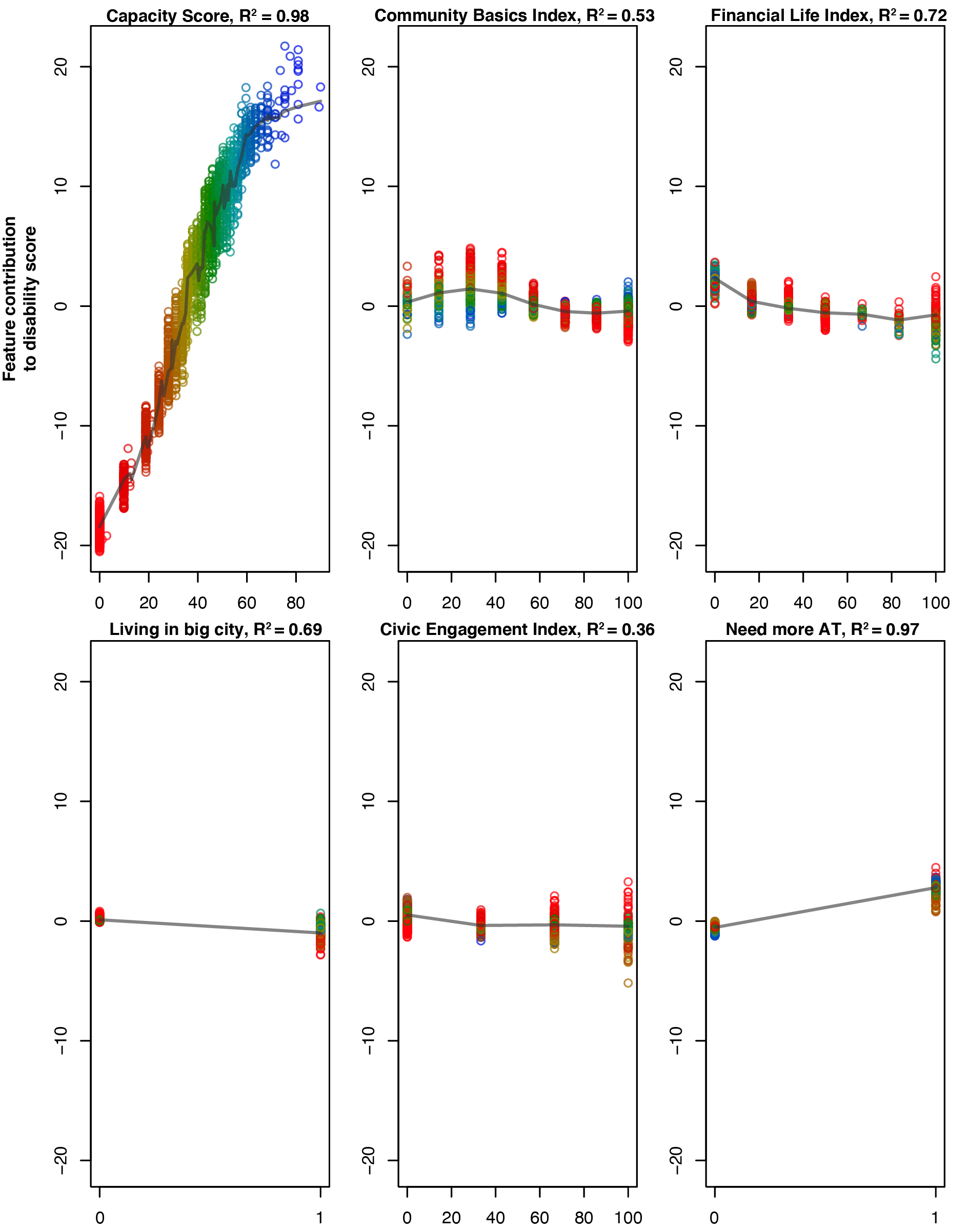


**Figure S1: Examples of feature contribution plots generated from the trained random forest model for Laos.** Each plot shows the cross-validated contribution of an individual feature (indicated in the plot title) to the disability score against the value of the feature. In this case, the plots are color-coded according to capacity score in order to identify potential interactions of capacity score and other variables. R-squared values indicate the goodness-of-fit of a k-nearest neighbor fit (gray line).


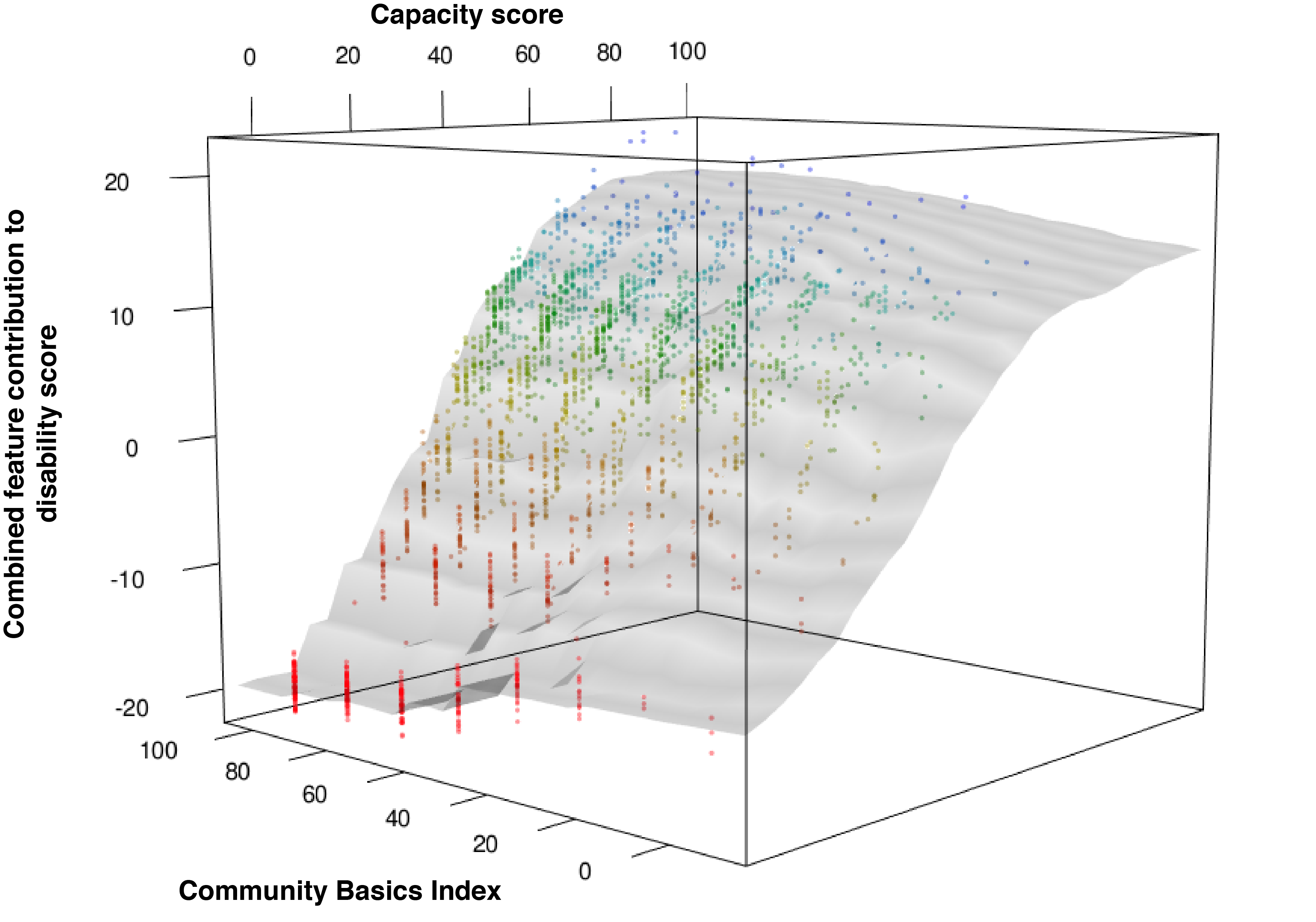


**Figure S2: A combined feature contribution plot of capacity score and community basics index reveals a significant interaction between the variables.** The plot shows the combined feature contribution of the capacity score and community basics index variables to the disability score. Evidence of interaction is determined by visual inspection and goodness-of-fit of a k-nearest neighbor surface in gray (R^2^ = 0.98). Each point represents an individual participant and is color-coded based on the capacity score (red = lower score, blue = higher score).

**Table S2: Minimum-parameter linear regression model for disability score in India.** R^2^ = 0.581.

|  |  | **Confidence Interval** | |  |
| --- | --- | --- | --- | --- |
| **Term** | **Value** | **Lower 95%** | **Upper 95%** | ***p-*value** |
| Intercept | 10.031 | 8.980 | 11.081 | <0.001 |
| Capacity Score | 0.833 | 0.807 | 0.860 | <0.001 |
| Sex | 1.020 | -0.071 | 2.111 | 0.067 |
| Age 30 to 49 | -0.591 | -1.840 | 0.658 | 0.353 |
| Age 50 or above | -0.680 | -2.176 | 0.816 | 0.373 |

**Table S3: Minimum-parameter linear regression model for disability score in Laos.** R^2^ = 0.494.

|  |  | **Confidence Interval** | |  |
| --- | --- | --- | --- | --- |
| **Term** | **Value** | **Lower 95%** | **Upper 95%** | ***p-*value** |
| Intercept | 18.577 | 17.449 | 19.705 | <0.001 |
| Capacity Score | 0.650 | 0.624 | 0.676 | <0.001 |
| Sex | -0.217 | -1.155 | 0.720 | 0.650 |
| Age 30 to 49 | -2.411 | -3.468 | -1.354 | <0.001 |
| Age 50 or above | -3.614 | -4.932 | -2.297 | <0.001 |

**Table S4: Minimum-parameter linear regression model for disability score in Tajikistan.** R^2^ = 0.604.

|  |  | **Confidence Interval** | |  |
| --- | --- | --- | --- | --- |
| **Term** | **Value** | **Lower 95%** | **Upper 95%** | ***p-*value** |
| Intercept | 4.634 | 3.816 | 5.452 | <0.001 |
| Capacity Score | 0.799 | 0.772 | 0.826 | <0.001 |
| Sex | 0.890 | -0.022 | 1.803 | 0.056 |
| Age 30 to 49 | 0.840 | -0.191 | 1.871 | 0.110 |
| Age 50 or above | 3.277 | 1.922 | 4.632 | <0.001 |
